# Clock‐drawing test: Normative data of three quantitative scoring methods for Chinese‐speaking adults in Shijiazhuang City and clinical utility in patients with acute ischemic stroke

**DOI:** 10.1002/brb3.1806

**Published:** 2020-08-27

**Authors:** Kai Shao, Fang‐Ming Dong, Shang‐Zun Guo, Wei Wang, Zhong‐Min Zhao, Yi‐Ming Yang, Pan‐Pan Wang, Jian‐Hua Wang

**Affiliations:** ^1^ Department of Graduate College Hebei Medical University Shijiazhuang China; ^2^ Department of Neurology Hebei General Hospital Shijiazhuang China; ^3^ Department of Graduate College Hebei North University Zhangjiakou China; ^4^ Department of Electromyography Hebei General Hospital Shijiazhuang China

**Keywords:** clock‐drawing test, ischemic stroke, normative data, scoring system, standardized value

## Abstract

**Objective:**

The clock‐drawing test (CDT) is a widely used screening tool for detecting cognitive decline. However, normative data for Chinese individuals are scarce. Our study aimed to provide standardized values for the three quantitative CDT scoring methods that were tailored for Chinese‐speaking adults in Shijiazhuang City and explore the discriminant validity of the CDT scores in patients with acute ischemic stroke.

**Methods:**

We conducted the CDT among 418 healthy individuals aged between 35 and 84 years. The CDT was administered and scored by five raters using the method derived from the Montreal Cognitive Assessment (MoCA), Rouleau's, and Babins’ scoring systems. The influence of age, education, and sex on the performance in the CDT was analyzed. Furthermore, 336 patients with acute ischemic stroke were enrolled to explore the discriminant validity of CDT scores.

**Results:**

In all three scoring systems, CDT scores were significantly correlated with age and years of education but not with sex. Normative data stratified for age and years of education were established. Interrater and intersystem reliability were high in our study. CDT total scores and subscores showed significant differences between stroke patients and healthy individuals.

**Conclusions:**

Our study provides CDT normative data using three quantitative scoring methods for Chinese‐speaking adults in Shijiazhuang City. Age and education level were the key factors that affected the CDT scores. CDT total scores and subscores provided good discriminant validity for patients with acute ischemic stroke.

## Graphical abstract

Age and education level were the key factors that affected the CDT scores. Normative data stratified for age and years of education were established. CDT total scores and subscores provided good discriminant validity for patients with acute ischemic stroke.

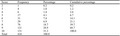

## 1 INTRODUCTION

With both the largest population and the largest aging population in the world, China has numerous patients being treated for cognitive impairment. It was reported that the prevalence of dementia was 5.14% in individuals aged 65 years or older (Jia et al., 2020) and the prevalence of mild cognitive impairment was 14.71% in people aged 60 years or older (Xue, Li, Liang, & Chen, 2018). This seriously threatens the health and quality of life of the elderly and places a huge burden on caregivers, families, and society (Wang et al., 2019). The high prevalence of dementia has raised the demand for an efficient and effective screening tool for detecting cognitive impairment (Borson et al., 2013). An ideal cognitive impairment screening test should be quick to administer, well tolerated and acceptable to patients, easy to score, and relatively independent of culture, language, and education (Shulman, 2000).

The clock‐drawing test (CDT) was originally developed as an instrument for attention and visual disorders, notably hemineglect syndrome (Battersby, Bender, Pollack, & Kahn, 1956; Shulman, 2000). In the current clinical setting, it is a valid screening tool for detecting cognitive decline. It is easy to administer with less than one minute of testing time, requires low cost, and is well tolerated by examinees. Ethnicity or language shows no significant effect on the CDT score, and education level may have a positive effect on it, but drawing a clock does not need high educational requirements (Borson et al., 1999). Patients with dementia of the Alzheimer type, Huntington's disease, Parkinson's disease, vascular disease, schizophrenia, and stroke have all demonstrated significant impairment on the CDT (Bozikas, Giazkoulidou, Hatzigeorgiadou, Karavatos, & Kosmidis, 2008). Several cognitive processes are necessary for drawing a clock. It entails concentration and attention to maintain the focus throughout the execution of the task (Noronha, Barreto, & Ortiz, 2018), requiring semantic memory to form the mental representation of a clock (Turcotte et al., 2018). Furthermore, performance on this test also depends on the executive functions that are responsible for planning, monitoring, inhibition, and correction of errors (Mazancova, Nikolai, Stepankova, Kopecek, & Bezdicek, 2017). In addition, the construction of the clock employs visuoconstructive skills that are required to draw the visual aspects of a clock (Caffarra et al., 2011). Thus, establishing standardized CDT values is of great clinical importance in the evaluation of cognition level.

There are different ways to administer the test, generally drawing on command, copying, or only reading the time from the clock. During clock reading, subjects must identify the time shown on a clock face and select the correct answer from three alternatives (multiple choice) (Bodner et al., 2004; Lam et al., 1998). In the copying version of the CDT, participants are shown an image of a clock and instructed to copy it (Nyborn et al., 2013; Royall, Cordes, & Polk, 1998). Compared to the reading and copying versions, the command version is more sensitive to cognitive impairment (Rouleau, Salmon, & Butters, 1996) and requires more visual and verbal memory, conceptualization, and language comprehension (Freedman et al., 1994). There are different processes of drawing the clock, making use of either predrawn or free‐drawn (Freedman et al., 1994; Lam et al., 1998; Rouleau, Salmon, Butters, Kennedy, & McGuire, 1992; Shulman, 2000). The use of a predrawn circle focuses on the clock‐drawing performance on number and hand placement (Tuokko, Hadjistavropoulos, Rae, & O'Rourke, 2000), whereas the use of a free‐drawn process allows the mistakes of drawing the circle to be detected. Besides, the time setting may differ (Pinto & Peters, 2009): 11:10, 2:45, 3:00, 8:20, 1:45, 10:10, with 11:10 being the most frequently used. It has been considered that “11:10” may be more sensitive to frontal dysfunction, including stimulus‐bound responses, as “10” has both concrete and abstract representations of the clock (Matsuoka et al., 2013). The 11:10 task is particularly useful because it requires visuospatial as well as the frontal inhibitory functions to not pull the hands toward number 10 instead of number 2 on the clockface (Lee, Kim, Choi, & Sohn, 2009).

Additionally, the scoring methods of the CDT are varied, including quantitative and qualitative analyses of the clock face, numbers, hands, and time settings. The qualitative analysis provides information about the participant's specific error types, but requires more time and training to be conducted properly. This trade‐off is one of the considerations in determining the utility of a scoring system in clinical settings with significant time constraints. Quantitative analysis is more commonly used in the clinical setting, and quantitative measures allow for faster, more practical scoring (Parsey & Schmitter‐Edgecombe, 2011). Thus, we chose to use quantitative measures to establish standardized values. Moreover, there are various scoring methods, ranging from simple to complex, and with different total point values, varying from three points (Lin et al., 2003) to thirty‐three points (Heinik, Solomesh, & Berkman, 2004). Therefore, the scoring methods have different levels of sensitivity and specificity. It is debatable which scoring method is the best, but the CDT has shown a high discriminative validity for screening purposes in moderate to severe dementia in the literature (Brodaty & Moore, 1997; Lessig, Scanlan, Nazemi, & Borson, 2008; Park, Jeong, & Seomun, 2018). Of these scoring systems, we selected one derived from the MoCA with a full score of three points (CDT3) (Kim, Jahng, Yu, Lee, & Kang, 2018), one referred to as Rouleau's method with a full score of ten points (CDT10) (Rouleau et al., 1992), and the other using Babins’ method with a full score of 18 points (CDT18) (Babins, Slater, Whitehead, & Chertkow, 2008). CDT3 and Shulman's system (Shulman, Shedletsky, & Silver, 1986) are the most studied and popular clock‐drawing methods, while CDT10 and CDT18 are more complex. However, to screen for mild cognitive impairment and dementia, complex scoring methods are recommended (Babins et al., 2008; Mazancova et al., 2017). The CDT normative data of Shulman's system have already been studied by Shanhu et al. (Shanhu et al., 2019), but the normative data of CDT3, CDT10, and CDT18 are limited in China.

In general, age and education have been considered to be the impact factors for CDT scores regardless of race or nationality (Merims, Ben Natan, Milawi, & Boguslavsky, 2018; Turcotte et al., 2018), but the effect of sex is not currently agreed upon (Shanhu et al., 2019; Sugawara et al., 2010). Normative data of the CDT have been published for the French (Turcotte et al., 2018), Israeli (Merims et al., 2018), Japanese (Sugawara et al., 2010), Portuguese (Santana, Duro, Freitas, Alves, & Simoes, 2013), American (Menon, Hall, Hobson, Johnson, & O'Bryant, 2012), and Czech (Mazancova et al., 2017), but the CDT normative data for the Chinese are scarce. A recent study (Shanhu et al., 2019) explored the influence of age, education, and sex on the CDT scores in an elderly sample in Hangzhou, China, but normative data in people aged <65 years were not reported. In addition, their diagnosis of normal cognitive function mainly depends on the Mini‐Mental State Examination (MMSE) without adjusting for education level, which is less rigorous.

This observational cross‐sectional study was developed in different phases with two goals to explore (a) the influence of age, education, and sex on the CDT scores of three quantitative scoring methods in a sample of Chinese‐speaking adults in Shijiazhuang and establish standardized values, and (b) the discrimination of CDT scores in patients with acute ischemic stroke and normal controls in the community.

## 2 STUDY 1: NORMATIVE DATA FOR CHINESE‐SPEAKING ADULTS IN SHIJIAZHUANG CITY

### 2.1 Methods

#### 2.1.1 Participants

Participants were recruited from adult individuals who registered to participate in a community service program for the early detection and management of dementia from two residential districts (the Yuxi community and the Liuying community) in Shijiazhuang, Hebei Province from June 2018 to October 2019. We recruited a convenience sample from the community service program that met the study criteria, with the goal of including a broad range of ages and education levels. The expert clinicians of the community hospital, who joined this study as study coordinators, interviewed each participant to screen for neurological and psychiatric disorders that could affect cognitive abilities. All participants were tested using a battery of neurological tests (Shao et al., 2020; Yang et al., 2020), including cognitive functions of memory, executive, information processing, attention, language as well as global cognition tests and functional and depression scales. The inclusion phase was based on the individual's performance on these tests that were specially built for this study, including the Chinese version of the MMSE, the Burns Depression Checklist (BDC), and the CDT. The inclusion criteria were as follows: (a) aged 35 years or older and Chinese as mother language; and (b) normal cognitive function, as defined by the MMSE, taking individuals’ education level into consideration. The cutoff points were 17/18 for people who had not gone to school, 20/21 for people who had informal literacy training or elementary education, and 23/24 for people who had middle school or higher education, because these cutoff values have been reported to ensure good sensitivity and specificity in detecting dementia in Chinese individuals (Katzman et al., 1988); and (c) capacity to perform activities of daily living intact, preferably confirmed by a caregiver. The exclusion criteria were as follows: (a) complaints of memory loss or other cognitive deficits or a diagnosis of dementia; (b) a history of neurological or psychiatric diseases, such as stroke, epilepsy, brain injury, brain tumor, anxiety, or depression (BDC > 10) (Liu et al., 2018); (c) a history of alcohol and/or drug abuse or psychotropic drug intake; and (d) those who could not complete the CDT due to visual or auditory abnormalities, communication or comprehension difficulties, or unwillingness to sign the informed consent form. Our study was approved by the Ethics Committee of Hebei General Hospital, and all individuals provided written informed consent before participating.

#### 2.1.2 Materials and procedures

The CDT was collected from MoCA and was conducted using command free‐drawing on a “5.5 × 7.5 cm” sheet of paper. The CDT instructions were as follows: “Please draw a clock. Put in all the numbers, and set the hands at 10 past 11.” After administering the test, we collected the CDT papers and distributed them among the five neurologists who scored them using three quantitative scoring methods. For experienced clinicians, the completion time of CDT3, CDT10, and CDT18 was less than 10 s, 30 s, and 50 s, respectively. The raters were blinded to participants’ names, ages, sex, and education levels. The CDT3 assigned one point each for drawing a closed circle, placing all expected numbers in their correct positions, and placing the clock hands correctly to reflect the requested time (Kim et al., 2018). With more specific criteria than the CDT3, the criteria for CDT10 were divided into three categories: integrity of the clock face (i.e., present without gross distortion) (CDT10.1, 2 points), presence and sequencing of the numbers (i.e., all present in the correct order and minimal errors in their spatial arrangement) (CDT10.2, 4 points), and presence and placement of the hands (i.e., hands are in the correct position and the size difference is respected) (CDT10.3, 4 points). For more details regarding the interpretation guidelines, see Rouleau et al. (Rouleau et al., 1992). The CDT18 is a modification of Freedman et al.’s 15‐point scale (Freedman et al., 1994). It was measured according to the five major components of clock drawing: integrity of the clock face (CDT18.1, 2 points); placement of the center (CDT18.2, 2 points); numbering (CDT18.3, 6 points); placement and size of the hands (CDT18.4, 6 points); and the overall clock gestalt (CDT18.5, 2 points) (Babins et al., 2008).

#### 2.1.3 Statistical analyses

Statistical analyses were performed using SPSS program 25.0 for Windows. Data were examined for normality, skewness, and range restriction. Age and education were normally distributed, while MMSE and CDT scores were negatively skewed. Measures of demographic characteristics and neuropsychological test scores were summarized with means and standard deviations (*SD*) for continuous measures and percentages for categorical variables. Spearman correlation for CDT3 and multiple regression analyses (stepwise method) for CDT10 and CDT18 was performed to explore the influence of demographic variables (age, education, and sex). The coefficient of determination (*R* ^2^) was calculated as the effect size of multiple regression analysis (Cohen, 1988). Mean (*SD*) and mean minus 1 *SD*, 1.5 *SD*, and 2 *SD* and percentile ranks were calculated and stratified according to the sociodemographic variables that affect CDT. Interrater reliability was analyzed using the intraclass correlation coefficient (ICC) with a two‐way mixed‐effects model to determine consistency among raters. Intersystem correlations of the CDT scoring systems were calculated using Spearman's rho.

### 2.2 Results

#### 2.2.1 Sample characteristics

A total of 418 eligible participants were enrolled in the study. Of these, 60.53% were men and 39.47% were women. The sociodemographic characteristics, MMSE, and CDT scores are detailed in Table 1. In our sample, CDT3 ranged from 1 to 3 points, with only 4.1% scoring ≤1 point. CDT10 ranged from 2 to 10 points, with only 2.6% scoring ≤4 points. CDT18 ranged from 2 to 18 points, with only 2.9% scoring ≤9 points. Frequency tables (listed in the Appendix 1) showed that more than 50% of the individuals in our sample scored near the ceiling across all the three scoring systems, which can be interpreted as a “ceiling effect,” that is, due to the low level of difficulty in the CDT, healthy individuals obtained relatively high scores (Mazancova et al., 2017).

**Table 1:** Descriptive statistics of demographic aspects, Mini‐Mental state examination (MMSE), and clock‐drawing test (CDT) for the total sample (*N* = 418)

	Mean	Standard deviation	Median	Range (min‐max)
Age (year)	63.03	7.79	64	35–84
Education (year)	9.32	3.14	9	0–18
MMSE	27.34	1.84	28	20–30
CDT3^a^	2.66	0.55	3	1–3
CDT10^b^	8.46	1.62	9	2–10
CDT18^c^	14.95	2.26	15	2–18

Abbreviation: MMSE, Mini‐Mental State Examination.

^a^ Clock‐drawing test scoring system derived from the Montreal Cognitive Assessment.

^b^ Clock‐drawing test scoring system of Rouleau et al. (1992).

^c^ Clock‐drawing test scoring system of Babins et al. (2008).

#### 2.2.2 Effect of age, education, and sex on CDT scores

Spearman correlations were performed on CDT3 scores and demographic variables. Spearman correlations showed that CDT3 scores were significantly negatively correlated with age (*r* (417) = −.158, *p* = .001) and significantly positively correlated with years of education (*r* (417) = .164, *p* = .001) but not significantly correlated with sex (*r* (417) = −.046, *p* = .346).

A series of multiple linear regression analyses (stepwise method) were conducted to determine the predictive value of sociodemographic variables on CDT performance and help establish proper stratification criteria. Sex was excluded from this multiple linear regression model due to its low effect sizes in the two scoring systems (CDT10: *p* = .995; CDT18: *p* = .978). The results indicated that age and education had a significant impact on the prediction of both CDT10 (*R^2^* = .080) and CDT18 (*R^2^* = .111). In CDT10, age was the only significant negative predictor of test performance (*β* = −0.111, *t* = −2.221, *p* < .05) and education was the only significant positive predictor of test performance (*β* = 0.226, *t* = 4.531, *p* < .001). In CDT18, age was the only significant negative predictor of test performance (*β* = −0.034, *t* = −2.367, *p* < .05) and education was the only significant positive predictor of test performance (*β* = 0.276, *t* = 5.620, *p* < .001).

#### 2.2.3 Standardized values stratified for age and years of education

According to the results above, age and years of education were the key sociodemographic variables and should be stratified in the normative data. A median split was conducted on the age variable in the sample producing equivalent sample size representation across groups, with 221 people in the stratification of 35–64 years old and 197 people in the stratification of 65–84 years old. Education was defined into three levels based on the Chinese educational system: no formal schooling or basic compulsory education, ≤9 years (*n* = 229); high school, 10–12 years (*n* = 135); and any university‐level education, ≥13 years (*n* = 54). Percentile ranks were displayed from 1st to 50th. Mean CDT scores and percentile ranks for CDT3, CDT10, and CDT18 are presented in Table 2.

**Table 2:** Mean CDT scores (mean, standard deviation) and percentile ranks for the CDT3, CDT10, CDT18 stratified for age and years of education (*N* = 418)

Age	Education	*N*	Mean (*SD*)	Mean −*SD*	Mean −1.5 *SD*	Mean −2 *SD*	Percentiles						
							1	2	5	10	15	25	50
CDT3^a^													
35–64	≤9	108	2.68 (0.54)	2.14	1.86	1.59	1.0	1.0	2.0	2.0	2.0	2.0	3.0
	10–12	77	2.75 (0.52)	2.23	1.97	1.72	1.0	1.0	1.9	2.0	2.0	3.0	3.0
	≥13	36	2.83 (0.38)	2.45	2.26	2.07	2.0	2.0	2.0	2.0	2.0	3.0	3.0
65–84	≤9	121	2.52 (0.60)	1.91	1.61	1.31	1.0	1.0	1.0	2.0	2.0	2.0	3.0
	10–12	58	2.71 (0.56)	2.15	1.87	1.59	1.0	1.0	1.0	2.0	2.0	2.8	3.0
	≥13	18	2.67 (0.49)	2.19	1.94	1.70	2.0	2.0	2.0	2.0	2.0	2.0	3.0
CDT10^b^													
35–64	≤9	108	8.38 (1.62)	6.76	5.96	5.15	3.1	4.0	5.0	6.0	6.4	8.0	9.0
	10–12	77	8.75 (1.40)	7.35	6.65	5.96	5.0	5.0	5.0	6.8	7.0	8.0	9.0
	≥13	36	9.17 (1.11)	8.06	7.51	6.95	6.0	6.0	6.0	7.7	8.0	9.0	9.5
65–84	≤9	121	7.99 (1.87)	6.12	5.19	4.25	2.2	3.0	4.1	5.0	6.0	7.0	8.0
	10–12	58	8.67 (1.46)	7.21	6.49	5.76	3.0	3.4	5.0	6.9	7.9	8.0	9.0
	≥13	18	8.67 (1.33)	7.34	6.68	6.01	6.0	6.0	6.0	6.9	7.0	7.8	9.0
CDT18^c^													
35–64	≤9	108	14.70 (2.22)	12.48	11.37	10.26	8.0	8.2	10.0	11.0	13.0	13.3	15.0
	10–12	77	15.68 (1.70)	13.98	13.12	12.27	11.0	11.6	12.0	13.0	13.7	15.0	16.0
	≥13	36	16.11 (1.49)	14.62	13.88	13.13	12.0	12.0	12.9	14.0	14.5	15.0	16.0
65–84	≤9	121	14.15 (2.65)	11.50	10.17	8.84	3.1	7.0	8.1	11.0	12.0	13.0	15.0
	10–12	58	15.34 (1.86)	13.48	12.55	11.62	10.0	10.2	11.0	13.0	13.0	14.0	16.0
	≥13	18	15.22 (1.99)	13.23	12.24	11.25	11.0	11.0	11.0	11.9	12.9	13.8	16.0

^a^ Clock‐drawing test scoring system derived from the Montreal Cognitive Assessment.

^b^ Clock‐drawing test scoring system of Rouleau et al. (1992).

^c^ Clock‐drawing test scoring system of Babins et al. (2008).

#### 2.2.4 Reliability analysis

The five raters were blinded to each other's assessments. Interrater reliability for all three CDT scoring systems of 22 protocols showed high concordance with the same significance level (*p* < .001), with slightly higher correlations for the more detailed scoring systems. The interrater reliability was measured using ICCs: CDT3 ICC = 0.716, 95% CI [0.560, 0.849], CDT10 ICC = 0.825, 95% CI [0.711, 0.912], CDT18 ICC = 0.832, 95% CI [0.721, 0.916]. Moreover, the scores of all three scoring systems were highly correlated with each other at the same significance level (*p* < .01), again with slightly higher agreement for the more detailed scoring systems. The intersystem reliability was measured using correlation coefficients: between CDT3 and CDT10: *r* = .729, between CDT10 and CDT18: *r* = .718, and between CDT3 and CDT18: *r* = .553.

## 3 STUDY 2: CLINICAL UTILITY OF CDT IN PATIENTS WITH ACUTE ISCHEMIC STROKE

### 3.1 Methods

#### 3.1.1 Participants

From April 2014 to December 2018, 336 patients with acute ischemic stroke at the Department of Neurology, Hebei General Hospital, were enrolled in our study. All participants were tested by the MMSE and CDT within three weeks after stroke onset. The inclusion criteria were as follows: (a) acute cerebral infarction; (b) aged 50 years or older; (c) evidence of no motor, sensory, visual, auditory, or language deficits that could impede the neuropsychological evaluations, such as hemiplegia, hemianopia, visuospatial neglect, and aphasia; and (d) willing to sign the informed consent form. The exclusion criteria were as follows: (a) other cerebrovascular diseases, such as transient ischemic attack (TIA) or hemorrhagic stroke; (b) preexisting dementia or other diseases that are known to affect cognition; and (c) a history of mental illness or depression (BDC > 10). All study purposes and procedures were thoroughly explained to each subject and/or caregivers, and informed consent was obtained.

#### 3.1.2 Materials and procedures

We divided the patients into three groups according to the MMSE scores stratified by education level: no cognitive impairment after stroke (No CI), minor cognitive impairment after stroke (Minor CI), and major cognitive impairment after stroke (Major CI). The No CI group included people with an MMSE score ≥ 20 (uneducated), MMSE score ≥ 25 (1–6 years of education), and MMSE score ≥ 28 (≥7 years of education). The Minor CI group included people with an MMSE score of 15–19 (uneducated), MMSE score of 18–24 (1–6 years of education), and MMSE score of 22–27 (≥7 years of education). The Major CI group included people with an MMSE score ≤ 14 (uneducated), MMSE ≤ 17 (1–6 years of education), and MMSE score ≤ 21 (≥7 years of education). These cutoff values have been reported to ensure good sensitivity and specificity in identifying normal cognition versus mild cognitive impairment and mild cognitive impairment versus dementia in the elderly Chinese individuals (Li, Jia, & Yang, 2016). Although the discriminant validity of the CDT total scores has been shown in previous studies (Babins et al., 2008; Grande et al., 2013; Rakusa, Jensterle, & Mlakar, 2018), the discriminant validity of the CDT subscores has not been sufficiently studied in the Chinese population. The CDT total scores and subscores were compared with 400 normal controls (NC) in Study 1 that were homogenous in age and education to stroke patients. In addition, to examine the validity of the CDT as a measure of cognitive function in patients with acute ischemic stroke, we investigated the correlation between CDT scores and MMSE, a widely used screening test for global cognitive function.

#### 3.1.3 Statistical analyses

One‐way analysis of variance (ANOVA) was used to analyze differences among groups. Comparisons among groups were performed with Bonferroni post hoc analysis. The partial eta‐squared index ($\eta_{p}^{2}$) was used as a measure of effect size. Effect size was classified into small (0.01 ≤ $\eta_{p}^{2}$ < 0.06), medium (0.06 ≤ $\eta_{p}^{2}$ < 0.14), and large ($\eta_{p}^{2}$ ≥ 0.14) according to the literature (Cohen, 1988). Spearman's correlation analysis was used to assess the relationship between the CDT and MMSE scores.

### 3.2 Results

#### 3.2.1 CDT analyses in NC, No CI, Minor CI, and Major CI groups

The mean age, education level, MMSE, and CDT performance of the NC (*n* = 400), No CI (*n* = 105), Minor CI (*n* = 180), and Major CI (*n* = 51) groups are given in Table 3. For age and education, one‐way ANOVA with Bonferroni post hoc tests showed no significant differences among the four groups (age, *p* = .137; education, *p* = .062). For the MMSE score, significant differences were found among groups, except for NC versus No CI (*p* = .876).

**Table 3:** Analysis of variance in the study groups in age, years of education, MMSE, and CDT performance (mean, standard deviation)

	NC *N* = 400 Mean (*SD*)	No CI *N* = 105 Mean (*SD*)	Minor CI *N* = 180 Mean (*SD*)	Major CI *N* = 51 Mean (*SD*)	*F*	*p*	$\eta_{p}^{2}$	*p*, Post hoc test (Bonferroni)					
								NC versus. No CI	NC versus Minor CI	NC versus Major CI	No CI versus Minor CI	No CI versus Major CI	Minor CI versus Major CI
Age	63.66 (6.69)	62.77 (7.20)	63.96 (8.01)	65.73 (11.19)	1.848	.137	0.008	1.000	1.000	.383	1.000	.126	.818
Education	9.03 (2.89)	8.54 (4.42)	8.39 (3.65)	8.08 (3.27)	2.451	.062	0.010	1.000	.198	.338	1.000	1.000	1.000
MMSE	27.29 (1.84)	27.62 (1.95)	24.07 (2.29)	17.71 (3.08)	394.33	<.001	0.618	.876	<.001	<.001	<.001	<.001	<.001
CDT3^a^	2.67 (0.56)	2.43 (0.66)	2.11 (0.76)	1.65 (0.91)	55.626	<.001	0.186	.006	<.001	<.001	.001	<.001	<.001
CDT10^b^	8.45 (1.64)	7.49 (1.84)	6.65 (2.27)	5.08 (2.58)	70.523	<.001	0.224	<.001	<.001	<.001	.002	<.001	<.001
CDT18^c^	14.93 (2.28)	13.39 (2.49)	12.28 (3.40)	9.55 (3.98)	80.906	<.001	0.249	<.001	<.001	<.001	.007	<.001	<.001
CDT10.1	1.73 (0.46)	1.66 (0.50)	1.48 (0.53)	1.25 (0.60)	20.256	<.001	0.077	1.000	<.001	<.001	.026	<.001	.023
CDT10.2	3.58 (0.67)	3.01 (1.13)	2.73 (1.24)	2.14 (1.47)	55.404	<.001	0.185	<.001	<.001	<.001	.113	<.001	.001
CDT10.3	3.14 (1.14)	2.82 (1.34)	2.44 (1.33)	1.69 (1.32)	29.519	<.001	0.108	.099	<.001	<.001	.071	<.001	.001
CDT18.1	1.61 (0.51)	1.56 (0.50)	1.41 (0.52)	1.16 (0.54)	15.558	<.001	0.060	1.000	<.001	<.001	.081	<.001	.014
CDT18.2	1.85 (0.40)	1.71 (0.55)	1.61 (0.60)	1.35 (0.82)	20.015	<.001	0.076	.085	<.001	<.001	.609	<.001	.009
CDT18.3	5.39 (0.86)	4.70 (1.43)	4.26 (1.64)	3.45 (1.77)	59.982	<.001	0.197	<.001	<.001	<.001	.024	<.001	<.001
CDT18.4	4.64 (1.36)	4.06 (1.63)	3.79 (1.70)	2.49 (1.94)	36.833	<.001	0.131	.003	<.001	<.001	.929	<.001	<.001
CDT18.5	1.43 (0.63)	1.35 (0.67)	1.22 (0.69)	1.10 (0.78)	6.817	<.001	0.027	1.000	.002	.005	.574	.148	1.000

Note

CDT10.1 = integrity of the clock face (2 points); CDT10.2 = presence and sequencing of the numbers (4 points); CDT10.3 = presence and placement of the hands (4 points); CDT18.1 = integrity of the clock face (2 points); CDT18.2 = placement of the center (2 points); CDT18.3 = numbering (6 points); CDT18.4 = placement and size of the hands (6 points); CDT18.5 = the overall clock gestalt (2 points); NC = normal control; No CI = no cognitive impairment after stroke; Minor CI = minor cognitive impairment after stroke; Major CI = major cognitive impairment after stroke; MMSE = Mini‐Mental State Examination.

^a^ Clock‐drawing test scoring system derived from the Montreal Cognitive Assessment.

^b^ Clock‐drawing test scoring system of Rouleau et al. (1992).

^c^ Clock‐drawing test scoring system of Babins et al. (2008).

The total scores in CDT3, CDT10, and CDT18 indicated good discriminative validity in the four groups. Bonferroni post hoc tests indicated that in the subscores of CDT10, most group differences were identified by CDT10.1, CDT10.2, and CDT10.3, except for CDT10.1 in discriminating NC versus No CI (*p* = 1.000), CDT10.2 in discriminating No CI versus Minor CI (*p* = .113), CDT10.3 in discriminating NC versus No CI (*p* = .099) and No CI versus Minor CI (*p* = .071). In the subscores of CDT18, most group differences were identified by CDT18.1, CDT18.2, CDT18.3, and CDT18.4. However, CDT18.5 only identified NC versus Minor CI (*p* = .002) and NC versus Major CI (*p* = .005). Furthermore, the differences between NC and No CI were not identified by CDT18.1 and CDT18.2, and the differences between the No CI and Minor CI were not identified by CDT18.1, CDT18.2, and CDT18.4. Only CDT18.3 identified all the differences among the four groups.

Regarding the error type reflected by the subscores of CDT10 and CDT18, such as clock face errors (CDT10.1 and CDT18.1), no differences were detected between NC and No CI. Regarding the clock number errors (CDT 10.2 and CDT18.3), CDT18.3 detected differences in the error of clock numbers between No CI and Minor CI, but CDT10.2 did not. This indicates that more detailed criteria may be more sensitive to the clock number error type. Regarding errors of clock hands (CDT10.3 and CDT18.4), no difference was found between No CI and Minor CI. CDT18.4 detected differences between NC and No CI, but CDT10.3 did not. This indicates that more detailed criteria may be more sensitive to the clock hands error type.

#### 3.2.2 Correlations between CDT and MMSE

The CDT scores obtained using each of the three scoring methods were significantly correlated with MMSE at the same significance level (*p* < .001): CDT3, *r* (335) = .380, CDT10, *r* (335) = .399, CDT18, *r* (335) = .422. Correlations between CDT and MMSE were significant, further demonstrating the validity of the CDT as a screening tool. Moreover, the correlation between CDT18 and MMSE was higher than that of the other two scoring systems, which indicated that CDT18 entailed more elements of global cognition.

## 4 DISCUSSION

This study established standardized values for the CDT of three quantitative scoring systems in a convenience sample of Chinese‐speaking adults in Shijiazhuang, across a broader spectrum of adult aging strata compared to an earlier published study only focusing on the Chinese elderly in Hangzhou (Shanhu et al., 2019). Individuals aged 35 years or older were enrolled in our study, which enlarged the applicability of CDT normative data, especially in a wide range of adults. Furthermore, the free‐drawn method entails more information about the clock face than the predrawn method used in the study by Shanhu et al., suggesting a more comprehensive evaluation in our study. To achieve a rigorous selection, we applied MMSE cutoffs taking individuals’ education level into consideration (Katzman et al., 1988) as one of the inclusion criteria, and all the expert clinicians in the community hospital were instructed to select patients who fulfilled the inclusion/exclusion criteria. The data derived from the study suggested that age and years of education were identified to have significant influences on the CDT performance regardless of which scoring method was applied. CDT scores were lower in older people or lower levels of education, whereas no effect was seen in sex. This is in accordance with other studies (Bozikas et al., 2008; Mazancova et al., 2017; Merims et al., 2018; Shanhu et al., 2019; Siciliano et al., 2016; Turcotte et al., 2018). Compared to developed countries, the education level of the population studied in our research is relatively low, with a mean education of 9.3 years. Eighteen participants had an education level less than or equal to three years of formal schooling, and two of them were illiterate. Therefore, our data may be useful in multinational and multicultural comparisons internationally. Regarding the impact of sex on CDT scores, there was no consensus. Norms of Japanese individuals (Sugawara et al., 2010) showed that sex had a significant effect on CDT scores, higher in women than in men, while norms of Portuguese individuals (Santana et al., 2013) found higher CDT scores in men than in women. Differences in the characteristics of the population, including culture, language, and various methodologies, could explain these discrepancies.

An advantage of our study is that the three quantitative scoring methods used in this research were selected from simple to complex methods. The analysis of process and errors afforded by the CDT is elegant for its simplicity as well as its capacity to appreciate complex cognitive operations (Grande et al., 2013). CDT3 is brief and easy to score. It is also used as part of more extensive cognitive function screening tests, such as the commonly used MoCA (Price et al., 2011) and Mini‐Cog scale (Borson, Scanlan, Brush, Vitaliano, & Dokmak, 2000) in clinical settings. CDT10 is usually based on a quantitative evaluation of specific errors of the clock face, numbers, and hands. It has been demonstrated to be valid in distinguishing individuals with mild Alzheimer dementia from normal elderly (Chiu, Li, Lin, Chiu, & Liu, 2008), but the use of CDT10 in patients with mild cognitive impairment should be treated with more caution due to the lower sensitivity and specificity for milder forms of cognitive impairment (Duro et al., 2019). CDT18 is more specific than the other two scoring systems. It entails more details than CDT10, including the assessment of the circle outline, number sequencing, and hand placement, especially the gestalt, center, and contour integrity of the clock face. CDT18 seems to be more informative and might have advantages in better discriminating individuals with mild cognitive impairment more likely to progress to dementia from those who will remain stable (Babins et al., 2008). Three widely used quantitative scoring methods enlarged the practicability of the normative data, justifying an adequate exploration of their psychometric properties and development of normative data for all of them.

Additionally, different statistical methods were applied to explore the influence of demographic variables on the CDT in our study. Spearman correlation was made on CDT3 instead of multiple linear regression because of the limited value range from 0 to 3 points. The value of CDT3 can only be 0, 1, 2, and 3, which can be regarded as discrete variables in quantitative variables or ordered variables in qualitative variables. When the age, education, and sex variables were put into linear regression, there was no evident linear trend in the scatter plot theoretically, since CDT3 had only 0, 1, 2, and 3 values. Therefore, Spearman correlations were performed for CDT3 and demographic variables. CDT10 and CDT18 have a wider value range; hence, multiple linear regressions were performed, which increased the efficacy of the data.

Due to the limited number of older people with relatively high levels of education, we made a median split for the stratification of age according to the literature (Bozikas et al., 2008; Mazancova et al., 2017; Sugawara et al., 2010) to ensure that each stratum had a moderate number of subjects when stratified for age and years of education simultaneously. Mean minus 1 *SD*, 1.5 *SD*, and 2 *SD* and percentile ranks calculated and stratified according to the sociodemographic data significantly influenced CDT scores. Percentile ranks are not affected by skewness and relevant for expressing scores because any given test score is within the population. We referred to the percentile ranks strata in the literature (Turcotte et al., 2018) and found that scores at percentile ranks of 1, 2, and 5 indicated pathological performances. Scores at the 10th percentile suggested mild impairment. Performances in the low average range corresponded to the 15th percentile. The first quartile and the median of the scores’ distribution in the normative sample were commensurate with the 25th and 50th percentiles, respectively.

Consistency among raters is necessary for neuropsychological tools. To raise the reliability of the CDT normative data, interrater and intersystem reliability were calculated. The interrater reliability indicated a very good agreement among the five raters according to literature, with ICC < 0.40 (poor), 0.40–0.59 (fair), 0.60–0.74 (good), and 0.75–1.00 (excellent) (Stienen et al., 2019). The ICCs of CDT10 and CDT18 were excellent and consistent with previous studies (Mazancova et al., 2017; South, Greve, Bianchini, & Adams, 2001). The ICC of CDT3 was good and lower than that of the other two scoring systems. This might be because it was less detailed and less accurate in the scoring criteria and thus more subjective during assessment. Furthermore, the reliability of the scoring systems was also proven by intersystem correlations. High correlations (*r* > .50, *p* < .001) (Cohen, 1992) were found in the three scoring systems. Owing to the simple nature of CDT3 and the complex nature of CDT18, the correlation coefficient between CDT3 and CDT18 was relatively low compared to that of between CDT3 and CDT10.

The discriminant validity of CDT subscores regarding error types has not been sufficiently studied in the Chinese population. Hence, we explored whether CDT total scores and subscores could differentiate patients with acute ischemic stroke and normal controls. ANOVA with Bonferroni post hoc tests indicated the total score and subscore differences among NC, No CI, Minor CI, and Major CI. According to the results, errors regarding the clock numbers were the most indicative items in differentiating individuals in the four groups. A study on qualitative CDT found that the inability to place evenly distributed gaps before 12, 3, 6, or 9 might be the most sensitive error during the early stages of dementia (Lee et al., 2009), which supports our findings. In addition, our results indicated that the more detailed criteria of clock numbers and clock hands in the CDT18 were more sensitive in differentiating individuals in the four groups than the criteria of the CDT10. This is in accordance with the previous points of view that more detailed criteria may be more sensitive (Mazancova et al., 2017; Parsey & Schmitter‐Edgecombe, 2011). Furthermore, the significant correlations between CDT and MMSE were consistent with the idea that CDT is valid for assessing cognitive dysfunctions (Sugawara et al., 2010).

Our study has some limitations. First, a convenience sample limits the ability to generalize the findings to the entire Chinese population. Ideally, a random sampling method would have been preferable, maximizing the representativeness of the sample, but we used a convenience sample with the goal of including a broad range of adult ages and education levels. We believe these results will be a valuable reference for clinicians and psychologists in China, especially in Shijiazhuang. Second, individuals older than 85 years were not present in our research because of the limited number of the elderly in the studied communities, which limits the application of the CDT normative data with this segment of the population. Finally, the inclusion criteria for the normal individuals were not very strict. Although we defined normal individuals by MMSE taking individuals’ education level into consideration, persons with very mild cognitive impairment could not be excluded completely.

## 5 CONCLUSION

Our study provides preliminary normative data of three quantitative CDT methods for Chinese‐speaking adults in Shijiazhuang City. Age and years of education influence CDT scores, which indicate that we must take these key factors into consideration when establishing standardized values. Good discriminant validity of CDT total scores and subscores has been obtained in patients with acute ischemic stroke. Future studies should establish the sensitivity and specificity of our normative data for detecting cognitive impairment in clinical populations.

## CONFLICT OF INTEREST

The authors declare no conflicts of interest.

## AUTHOR CONTRIBUTIONS

Jian‐Hua Wang made a substantial contribution to the design of the work. Kai Shao wrote the first draft of the manuscript. Fang‐Ming Dong, Wei Wang, Zhong‐Min Zhao, and Yi‐Ming Yang collected the data and analyzed it in collaboration with Kai Shao. Shang‐Zun Guo, Zhong‐Min Zhao, Yi‐Ming Yang, and Pan‐pan Wang helped edit and revise it critically for important intellectual content. All authors approved the final submitted version of the manuscript.

### Peer Review

The peer review history for this article is available at https://publons.com/publon/10.1002/brb3.1806.

## Data Availability

The data that support the findings of this study are available from the corresponding author upon reasonable request.

## Appendix 1

**Table A1:** Frequency table of CDT3

Score	Frequency	Percentage	Cumulative percentage
1	17	4.1	4.1
2	107	25.6	29.7
3	294	70.3	100.0
Total	418	100.0	

**Table A2:** Frequency table of CDT10

Score	Frequency	Percentage	Cumulative percentage
2	1	0.2	0.2
3	4	1.0	1.2
4	6	1.4	2.6
5	17	4.1	6.7
6	31	7.4	14.1
7	29	6.9	21.1
8	78	18.7	39.7
9	121	28.9	68.7
10	131	31.3	100.0
Total	418	100.0	

**Table A3:** Frequency table of CDT18

Score	Frequency	Percentage	Cumulative percentage
2	1	0.2	0.2
7	4	1.0	1.2
8	3	0.7	1.9
9	4	1.0	2.9
10	6	1.4	4.3
11	12	2.9	7.2
12	21	5.0	12.2
13	39	9.3	21.5
14	50	12.0	33.5
15	73	17.5	51.0
16	99	23.7	74.6
17	77	18.4	93.1
18	29	6.9	100.0
Total	418	100.0	
